# Assessment of LAA Strain and Thrombus Mobility and Its Impact on Thrombus Resolution—Added-Value of a Novel Echocardiographic Thrombus Tracking Method

**DOI:** 10.1007/s13239-022-00629-z

**Published:** 2022-05-13

**Authors:** Pedro Morais, Dominik Nelles, Vivian Vij, Baravan Al-Kassou, Marcel Weber, Georg Nickenig, Jan Wilko Schrickel, João L. Vilaça, Alexander Sedaghat

**Affiliations:** 1 2Ai – School of Technology, IPCA, Barcelos, Portugal; 2 Med. Klinik und Poliklinik II, Herzzentrum Bonn, Universitätsklinikum Bonn, Bonn, Germany

**Keywords:** Left atrial appendage, LAA thrombus, LAA longitudinal strain, Thrombus mobility, Image tracking

## Abstract

**Purpose:**

The mobility of left atrial appendage (LAA) thrombi and changes hereof under anticoagulation may serve as a marker of both risk of embolism and efficacy of treatment. In this study, we sought to evaluate thrombus mobility and hypothesized that LAA dynamics and thrombus mobility could serve as a baseline marker of thrombus dissolvability.

**Methods:**

Patients with two-dimensional transesophageal echocardiographic images of the LAA, and with evidence of LAA thrombus were included in this study. Using a speckle tracking algorithm, functional information from the LAA and thrombi of different patients was computed. While the LAA motion was quantified through the longitudinal strain, thrombus mobility was evaluated using a novel method by directly tracking the thrombus, isolated from the global cardiac motion. Baseline characteristics and echocardiographic parameters were compared between responders (thrombus resolution) and non-responders (thrombus persistence) to anticoagulation.

**Results:**

We included 35 patients with atrial fibrillation with evidence of LAA thrombi. Patients had a mean age of 72.9 ± 14.1 years, exhibited a high risk for thromboembolism (CHA2DS2-VASc-Score 4.1 ± 1.5) and had moderately reduced LVEF (41.7 ± 14.4%) and signs of diastolic dysfunction (*E*/*E*′ = 19.7 ± 8.5). While anticoagulation was initiated in all patients, resolution was achieved in 51.4% of patients. Significantly higher LAA peak strain (− 3.0 ± 1.3 vs. − 1.6 ± 1.5%, *p* < 0.01) and thrombus mobility (0.33 ± 0.13 mm vs. 0.18 ± 0.08 mm, *p* < 0.01) were observed in patients in whom thrombi resolved (i.e. responders against non-responders). Receiver operating characteristic (ROC) analysis revealed a high discriminatory ability for thrombus mobility with regards to thrombus resolution (AUC 0.89).

**Conclusion:**

Isolated tracking of thrombus mobility from echocardiographic images is feasible. In patients with LAA thrombus, higher thrombus mobility appeared to be associated with thrombus resolution. Future studies should be conducted to evaluate the role of the described technique to predict LAA thrombus resolution or persistence.

**Supplementary Information:**

The online version contains supplementary material available at 10.1007/s13239-022-00629-z.

## Introduction

Non-valvular atrial fibrillation (NVAF) is an established precipitator of cardioembolic stroke and peripheral embolism.^19^ Thrombus formation is caused by impaired left atrial hemodynamics and stasis is the main source of embolic complications in the context of AF.^7^ Hereby, most intracardiac thrombi in NVAF patients are found within the left atrial appendage (LAA).^18^ In clinical routine, different treatment options are available to prevent or treat LAA thrombus formation and the risk of stroke. While direct oral anticoagulants have replaced vitamin k-antagonists as the standard of care in most patients with NVAF,^9,17,29^ inefficacy of thrombus resolution or persistence of thrombus remains a clinical issue, especially as these patients appear to be at an increased risk for ischemic endpoint.^24^ While the change in medical treatment strategy is the most intuitive strategy for LAA thrombus,^24^ percutaneous LAA occlusion to “trap” the thrombus can also be performed in this scenario.^27^ Whereas the safety and efficacy of the different treatment options have been elucidated in previous studies,^16,24,27^ relevant clinical and echocardiographic predictors for the determination of LAA thrombus persistence or resolution are less well established. In this study, we introduce a novel concept to evaluate thrombus mobility and hypothesize that LAA dynamics and thrombus mobility are potential clinical indicators of thrombus persistence and/or resolution.

## Materials and Methods

### Study Population

A total of 35 patients who underwent elective two-dimensional transesophageal echocardiography (TEE) between the years 2013 and 2019 at the Heart Center of the University Hospital of Bonn (Bonn, Germany), and in whom the diagnosis of LAA thrombus was made, were retrospectively reviewed for eligibility. Only patients in whom anatomical tracking of the LAA was successful and in whom data quality was sufficient were included in the study. Patients with degenerated heart valve prostheses, post-mitral valvuloplasty and device-related thrombi as well as those after surgical LAA ligation were excluded. Moreover, only patients with solid LAA thrombus, which could be delineated in the B-Mode images were included. After diagnosis of LAA thrombus, all patients were treated with novel oral anticoagulants or Vitamin-K antagonists. Relevant clinical and demographic characteristics, antiplatelet and antithrombotic therapy were evaluated at baseline and follow-up.

### Echocardiography Studies

To evaluate thrombus resolution, patients underwent at least one follow-up TEE at discretion of the treating physician, to confirm thrombus resolution or persistence. All patients were divided into two groups, responder (thrombus resolution, i.e. no thrombus found at follow-up TEE) or non-responder (thrombus persistence or growth, i.e. thrombus found at follow up TEE).^24^ Echocardiographic parameters including left ventricular function and left atrial dimensions were obtained in accordance with current guidelines.^14^ We defined LAA thrombus as an organized mass within the LAA, which was visualized in at least two perpendicular planes during echocardiography.^24^ All echocardiograms were obtained using latest generation echocardiography machines, i.e. GE Vivid E95 and Vivid E9 (GEVingmed, Horten, Norway) and Philips iE33 and EPIQ CVx (Philips, Bothell, WA, USA). The LAA thrombus registry, from which the data was extracted, was approved by the local ethics committee at the University Hospital of Bonn (local ethics committee registration number: 188/2013).

### Anatomical Tracking

To assess the clinical potential of the motion information from both LAA and the thrombi itself, the LAA strain and thrombus mobility values were compared at the baseline for responder and non-responder groups.

**Figure 1:**
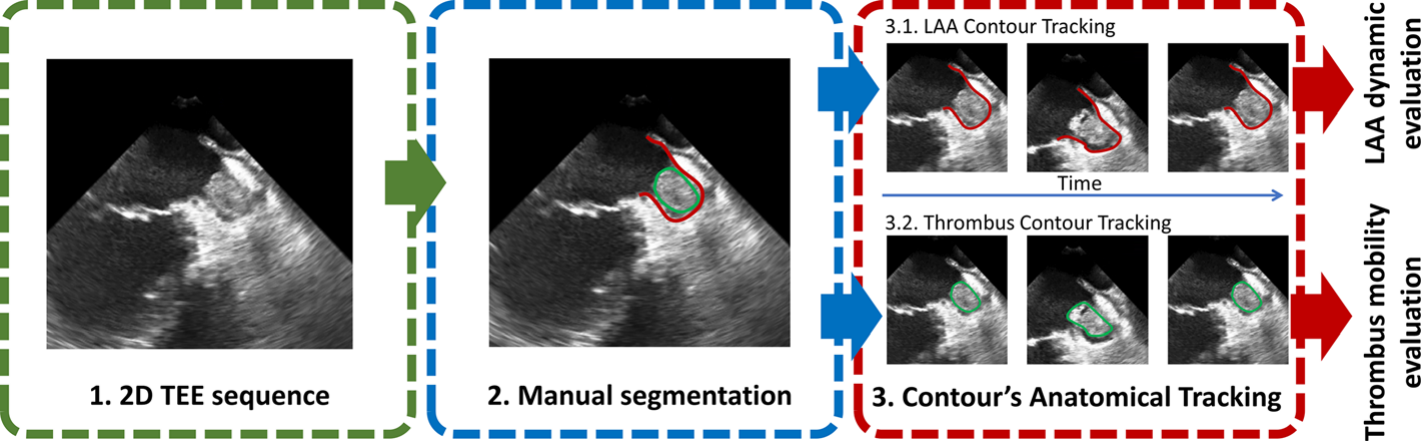
Overview of the anatomical tracking strategy.

One operator started by manually segmenting both anatomies (Fig. 1, block 2). The segmentations were performed at the end-diastolic phase and then automatically tracked throughout the remaining cycle using the Medical Imaging Tracking Toolbox (MITT)^26^ (Fig. 1). The MITT uses an anatomically constrained affine optical flow to track speckle patterns in echocardiographic images.^10^ The tracking is computed in a single cardiac cycle and the motion between consecutive frames is computed using optical flow. Instead of full image tracking, a set of regions-of-interest (ROI) anatomically defined around each contour were used. This localized strategy allows a reduction of the computational power of the method and makes it less sensitive to global variation of the image appearance, ultimately, increasing the robustness of the tracking around the target structure. The LAA and thrombus contours were discretized into a total of 80 and 40 points, respectively, and then propagated using a two-stage tracking approach with global and localized affine models to compensate fast LAA contraction and to refine the result to the anatomy. Note that both LAA and thrombus contours were tracked independently using the same strategy (see some tracking results in Supplementary Material I–V). Concerning our two-stage approach, while the initial global affine model will capture the global cardiac motions (e.g. full motion of the entire cardiac muscle) the localized models will target the small anatomical adaptation of each structure (e.g. LAA contraction). A pairwise tracking strategy with bidirectional propagation scheme (i.e., the tracking result is propagated half of the frames forward, and half backward) was applied between consecutive frames of the 2D sequence.

Concerning the tracking parameters, each ROI was defined as a window with a radius of ~ 6 × 6 mm and ~ 3 × 3 mm for the LAA and the thrombus, respectively. The Gaussian kernel’s sigma was ~ 0.4 × 0.4 mm with localized support of 25%. Constant and periodic boundaries were used for the LAA and thrombus contours, respectively. Out-of-field-of-view regularization was not used.

A customized and non-commercial software based on Matlab (MathWorks, Natick, MA, USA) was developed and used for the manual segmentation and tracking analysis. All results were checked, and corrections of the initial contour were performed if deemed necessary.

#### LAA Strain

After the LAA tracking throughout the full cardiac cycle, the longitudinal strain curve was computed based on the distance between consecutive points of the LAA contour (at each time frame) relatively to the original spacing at the end-diastolic phase, as described in Ref. 3.

Finally, the minimal and maximal peak strain values was computed for each patient, as previously described.^11,13^ Hereby, the minimal and maximal peak strains represent the highest LAA contraction (i.e. smallest volume) and relaxation (i.e. largest volume), respectively.

#### Evaluation of Thrombus Mobility

**Figure 2:**
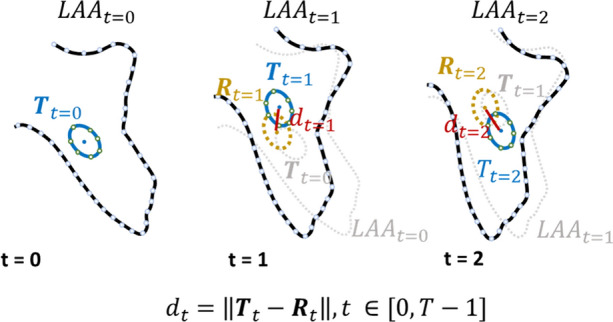
Overview of the thrombus mobility quantification strategy. For each time frame *t* (defined between 0 and *T* − 1, where *T* is the maximum number of frames), the LAA (blue line) and thrombus (black line, represented as *T*) contours are shown, as well as, their contour in the previous frame (gray line). The yellow line (contour R) represents the rigid component of the thrombus’s dynamics. ${d}_{t}$ represents the Euclidean distance between contours’ centroids for the time *t*.

To quantify thrombus mobility, a novel methodology was explored (Fig. 2). Instead of individual tracking, we fused the motion components of both LAA and thrombus. Thus, at each time frame, a double step tracking strategy is applied, namely: (i) creation of an intermediate tracking result (yellow contour in Fig. 2) where the thrombus contour is propagated from the previous frame (gray contour in Fig. 2) based on the LAA tracking result only; (ii) estimation of the local thrombus dynamics, by evaluating the spatial differences between the intermediate result (step 1, yellow contour in Fig. 2) and the thrombus tracking result computed as described in “Anatomical Tracking” (blue contour in Fig. 2).

In the first step, the LAA tracking result was used as a reference to quantify the influence of the external components at the thrombus positions. Thus, by assuming full compliance of the thrombus with the LAA boundaries, an intermediate result where all external motion components were removed is created. For that, based on the LAA affine motion fields defined at the LAA contour’s point, an interpolation strategy was used to estimate the influence of the external components at the thrombus positions (i.e. thrombus contour), as described in Ref. 21. In detail, using as anchors the LAA motion fields, the new motion vectors at each thrombus contour’s positions are interpolated from all anchors weighted by their Euclidean distances to the thrombus position. To guarantee a smooth result, a strategy with global support (i.e. where all anchors are used) is applied. Thus, the result of this interpolation strategy is an intermediate position (represented as yellow in Fig. 2 for *t *= 1 and *t* = 2), representing the thrombus position in case of its full compliance with the external movements (i.e. neglecting the specific motions of the thrombus). Please note that in cases of full compliance (i.e. if the thrombus has no specific motion), both blue (i.e. thrombus tracking result) and yellow contours (i.e. thrombus interpolated position) in Fig. 2 must be superimposed.

Then, in the second step, the specific thrombus dynamic was quantified by computing the Euclidean distance (in mm) between the thrombus contour’s centroid at the intermediate result and the thrombus tracking result at the current frame (computed as described in “Anatomical Tracking”). For each case, the mean and maximum Euclidean distances were calculated. For the purpose of this analysis, the study population was also divided based on the median mobility. Hereby, lower mobility was defined as a value below the median, while higher mobility was defined as values above the median.

### Statistical Analysis

Statistical analysis was performed using IBM Statistics version 20 (IBM Corp., Armonk, NY) and Microsoft Excel 365 (Microsoft, Redmond, WI). Variables were expressed as mean ± standard deviation (SD) or number (percentage) where appropriate. The D’Agostino-Pearson test was used to test the normality of the data. Two-tailed *p* values were calculated and considered to be significant if ranging below 0.05. Moreover, the receiver operating characteristic (ROC) curves and the area under the curve (AUC) were computed to determine the variables with best predictive value for thrombus resolution. Comparisons between the responder and non-responder groups were performed using Student’s *t*-test. For categorical data, Fisher’s exact test was calculated. Concerning the strain analysis, both responder and non-responder groups were compared through an unpaired t-test in terms of minimum, maximum and absolute maximum strain value. In both groups, the thrombus dynamics were also compared as described in “Evaluation of Thrombus Mobility”. An unpaired t-test was used to confirm statistically significant differences between groups. Moreover, a multiple regression analysis was computed to confirm if LAA mobility predicts thrombus resolution after adjusting for LA pressure (*E*/*e*′), LAA volume and screening time between TEE exams. As a final experiment, both Student’s *t*-test and Fisher’s exact test were computed to statistically compare the high and low mobility groups.

## Results

### Patient Characteristics

The mean age of the study cohort was 72.9 ± 14.1 years and 54.3% were male. All patients presented a history of AF with paroxysmal AF in 51.4% of cases; however, 14.3% of patients had documented sinus rhythm at diagnosis of thrombus. The population exhibited a high risk for thromboembolism (CHA2DS2-VASc 4.1 ± 1.5) and a moderate risk for bleeding (HAS-BLED score—2.8 ± 1.0). Of the entire cohort, 51.4% (18/35) showed complete thrombus resolution after 99.2 ± 71.8 days while LAA thrombus persisted in 48.6% (17/35) (non-responders). Baseline characteristics according to thrombus resolution are shown in Table 1.

**Table 1:** Clinical baseline characteristics.

Parameter	Total	Responder	Non-Responder	*p*
*N*	35	18	17	
Age (years)	72.9 ± 14.1	71.0 ± 15.4	74.8 ± 12.7	0.43
Men (abs./%)	19 (54.3)	11 (61.1)	8 (47.1)	0.51
eGFR (mg/dL)	50.5 ± 18.7	49.4 ± 20.8	51.7 ± 16.7	0.72
Renal insufficiency^a^ (abs./%)	7 (20.0)	3 (16.7)	4 (23.5)	1.00
Type of AF				
Paroxysmal (abs./%)	18 (51.4)	7 (38.9)	11 (64.7)	0.18
Persistent (abs./%)	14 (40.0)	9 (50.0)	5 (29.4)	0.31
Permanent (abs./%)	3 (8.6)	2 (11.1)	1 (5.9)	1.00
Diabetes (abs./%)	5 (14.3)	2 (11.1)	3 (17.6)	0.29
Hypertension (abs./%)	31 (88.6)	17 (94.4)	14 (82.3)	0.34
Coronary artery disease (abs./%)	21 (60.0)	10 (55.6)	11 (64.7)	0.73
Prior MI (abs./%)	16 (45.7)	8 (44.4)	8 (47.1)	1.00
Prior Stroke (abs./%)	5 (14.3)	3 (16.7)	2 (11.8)	1.00
Prior peripheral embolism (abs./%)	1 (2.9)	0 (0.0)	1 (5.9)	0.49
Cardiomyopathy (abs./%)	11 (31.4)	7 (38.9)	4 (23.5)	0.32
Ischemic (abs./%)	9 (25.7)	5 (27.8)	4 (23.5)	1.00
Dilatative (abs./%)	2 (5.7)	2 (11.1)	0 (0.0)	0.49
CHA2DS2-VASc score	4.1 ± 1.5	4.2 ± 1.6	4.1 ± 1.4	0.83
HAS-BLED score	2.8 ± 1.0	2.9 ± 0.9	2.7 ± 1.1	0.49
Medical therapy at baseline				
NOAC (abs./%)	16 (45.7)	6 (33.3)	10 (58.8)	0.18
Any OAC (abs./%)	25 (71.4)	10 (55.6)	15 (88.2)	0.06

^a^eGFR < 30 mL/min

### Echocardiographic Parameters

Overall, the selected patients revealed a moderately impaired left ventricular ejection fraction of 41.7 ± 14.4% and signs of diastolic dysfunction with an *E*/*E*′ of 19.7 ± 8.5. The time difference between the TEE at baseline and the follow-up was 51.0 ± 14.4 days without differences between the responder and non-responder groups (p = 0.65). The mean diastolic volume of the left atrium and LAA was 76.3 ± 33.2 mL and 6.8 ± 3.6 mL, respectively. Mean thrombus size was 1.2 ± 0.6cm × 2.0 ± 1.3 cm at diagnosis. The majority of patients of the patients (94.3%) exhibited spontaneous echo contrast, with 48.6% revealing dense spontaneous echo contrast in the left atrium at the time of thrombus diagnosis. The minimum and maximum peak LAA longitudinal strain was − 2.3 ± 1.6% and 3.2 ± 2.6%, while thrombus mobility was 0.25 ± 0.13 mm. Of interest, no significant differences in clinical baseline data were observed between patients with or without thrombus resolution.

Regarding echocardiographic parameters, statistically significant differences were found between the two groups in terms of LAA volume (4.9 ± 1.2 mL vs. 8.8 ± 4.2 mL, *p* < 0.01), and LAA peak emptying velocity (31.2 ± 10.8 vs. 17.5 ± 6.5 cm/s, *p* < 0.01). Table 2 depicts an overview of the extracted echocardiographic parameters at baseline for responder and the non-responder groups.

**Table 2:** Echocardiographic baseline characteristics.

Parameter	Total	Responder	Non-Responder	*p*
*N*	35	18	17	
Left ventricular end-diastolic volume (mL)	135.7 ± 77.2	130.1 ± 79.3	141.7 ± 76.9	0.67
Left ventricular end-systolic volume (mL)	87.8 ± 67.1	87.3 ± 62.3	88.3 ± 74.0	0.97
Left ventricular end-diastolic diameter	6.0 ± 1.3	6.2 ± 1.3	5.8 ± 1.2	0.50
Left ventricular ejection fraction (%)	41.7 ± 14.4	40.0 ± 10.1	43.5 ± 18.0	0.45
Left atrial end-diastolic volume (mL)	76.3 ± 33.2	83.25 ± 36.5	68.8 ± 28.5	0.20
E-wave (cm/s)	94.5 ± 40.4	93.9 ± 45.4	95.2 ± 36.3	0.93
E’-wave (cm/s)	4.9 ± 2.5	4.9 ± 2.9	5.0 ± 2.9	0.90
*E*/*E*′	19.7 ± 8.5	16.4 ± 3.5	25.4 ± 11.6	0.02
*LAA specific parameters*				
LAA Volume (mL)	6.8 ± 3.6	4.9 ± 1.2	8.8 ± 4.2	<0.01
LAA thrombus	1.6 ± 0.6	1.8 ± 0.7	1.4 ± 0.5	0.10
Minimum diameter (mm)	1.2 ± 0.6	1.3 ± 0.8	1.1 ± 0.3	0.34
Maximum diameter (mm)	2.0 ± 1.0	2.2 ± 0.9	1.7 ± 1.0	0.12
Time till resolution (days)	99.2 ± 71.8		–	–
Peak emptying velocity (cm/s)	24.5 ± 11.2	31.2 ± 10.8	17.5 ± 6.5	<0.01
Maximum LAA longitudinal strain (%)	3.2 ± 2.6	2.9 ± 2.5	3.5 ± 2.7	0.50
Minimum LAA longitudinal strain (%)	− 2.3 ± 1.6	− 3.0 ± 1.3	− 1.5 ± 1.5	<0.01
Mean Thrombi mobility (mm)	0.25 ± 0.13	0.33 ± 0.13	0.18 ± 0.08	<0.01
Spontaneous Echo Contrast (abs./%)	33 (94.3)	17 (94.4)	16 (94.1)	1.00
Mitral regurgitation (abs./%)	33 (94.3)	17 (94.4)	16 (94.1)	1.00
≥ Moderate (abs./%)	21 (60.0)	10 (55.6)	11 (64.7)	0.73
Mitral stenosis (abs./%)	6 (17.1)	3 (16.7)	3 (17.6)	1.00
≥ Moderate (abs./%)	2 (5.7)	1 (5.6)	1 (5.9)	1.00
Screening time between TEE exams	51.0 ± 14.4	52.1 ± 19.0	49.8 ± 7.3	0.65

**Figure 3:**
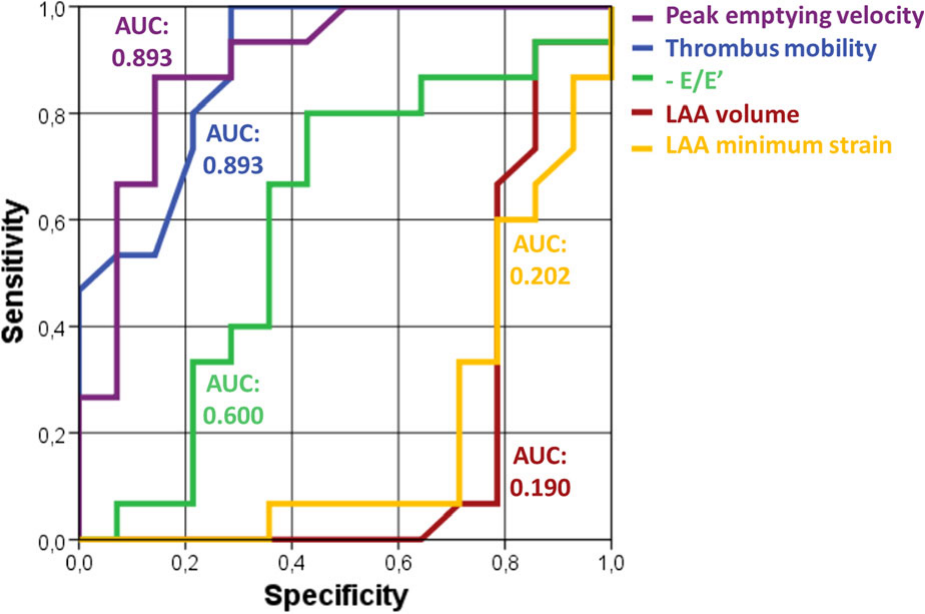
ROC curve for the thrombus mobility, LAA minimum strain, LA pressure (*E*/*e′*), LAA volume and peak emptying velocity in terms of thrombus resolution. Note that due to their theoretic inverse relationship with thrombus resolution the variables *E/e′*, LAA volume and LAA minimum strain were inversed prior to computing the c-statistic.

Figure 3 and Table 3 depict the ROC curve and AUC results, respectively, for the thrombus mobility, LAA minimum strain, LA pressure (*E*/*e*′), LAA volume and peak emptying velocity. The highest AUC was found for the thrombus mobility (AUC = 0.893) and peak emptying velocity (AUC = 0.893) parameters.

**Table 3:** AUC values with 95% confidence intervals (CI) for the thrombus mobility, LAA minimum strain, LA pressure (*E*/*e*′), LAA volume and peak emptying velocity in terms of thrombus resolution prediction.

Parameter	AUC (95% CI)	*p* value
Thrombus mobility	0.893 (0.776–1.000)	< 0.001
LAA minimum strain	0.798 (0.625–0.970)	0.006
*E*/*e*′	0.600 (0.381–0.819)	0.359
LAA volume	0.810 (0.621–0.998)	0.005
Peak emptying velocity	0.893 (0.771–1.000)	< 0.001

Note that due to their theoretic inverse relationship with thrombus resolution the variables *E*/*e*′, LAA volume and LAA minimum strain were inversed prior to computing the c-statistic

### Strain Analysis

**Figure 4:**
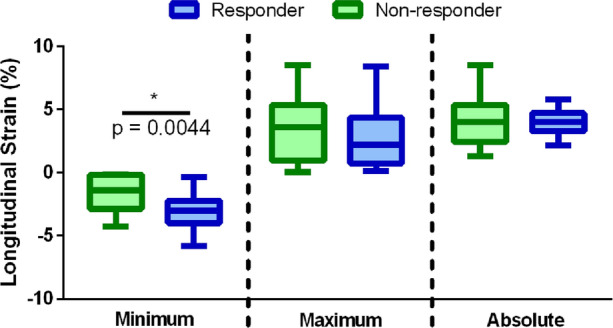
Minimal, maximal and absolute maximal peak strain results for both responder (blue) and non-responder (green) groups. *p < 0.05, unpaired *t*-test between the responder and non-responder groups.

**Figure 5:**
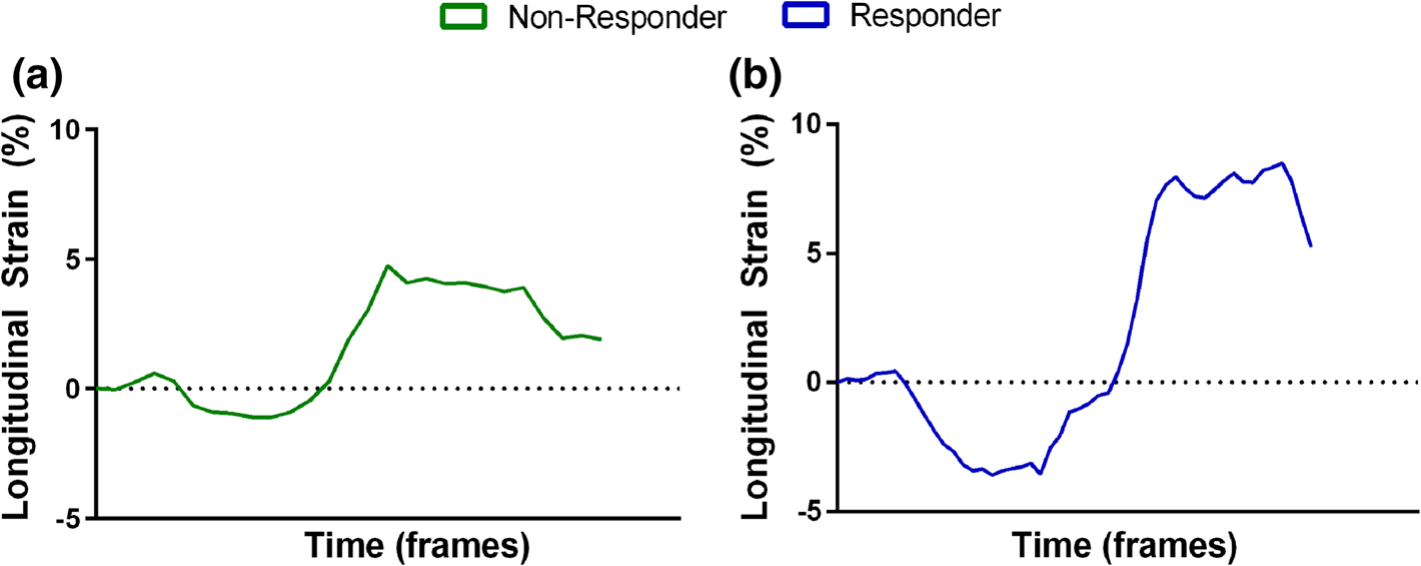
Representative example of one strain curve result for the responder (blue) and non-responder (green) groups.

**Figure 6:**
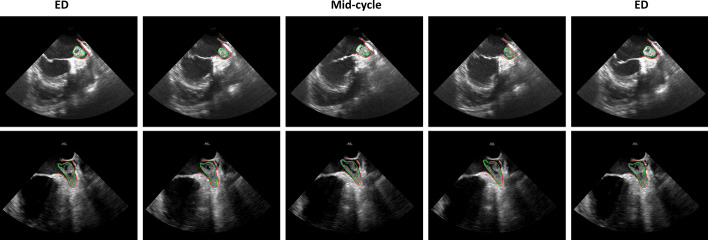
Representative tracking results.

Figure 4 depicts strain analysis between both responder and non-responder groups. The responder group showed a higher LAA contractibility with statistically significant differences against the non-responder group for the minimal peak LAA strain (− 3.0 ± 1.3 vs. − 1.6 ± 1.5%, *p* < 0.01). Examples of strain curves for responder and non-responder groups are also presented in Fig. 5. Representative examples of the tracking result are presented in Fig. 6 and in the Supplementary Material I–V.

### Thrombus Mobility Analysis

**Figure 7:**
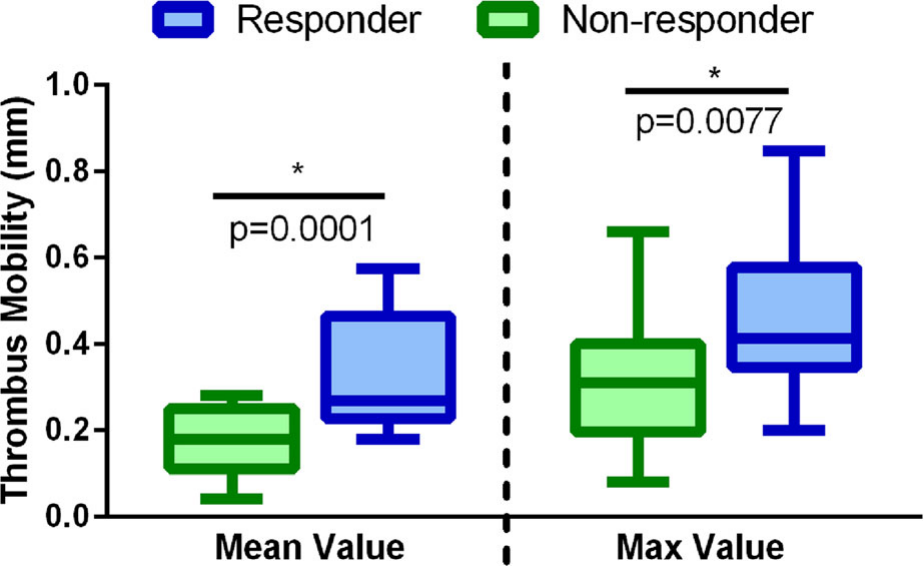
Quantification of the thrombus mobility in the responder (blue) and non-responder (green) groups. *p < 0.05, unpaired t-test between the responder and non-responder groups.

Figure 7 shows the thrombus mobility differences for responder/non-responder groups (described in “Evaluation of Thrombus Mobility”). Higher mobility was found in cases of thrombus resolution with statistically significant differences compared to the cases of thrombus persistence (0.33 ± 0.13 vs. 0.18 ± 0.08 mm, *p* = 0.0001). No statistically significant differences were found in the multiple regression analysis (*p* > 0.05, further details in supplementary material VI). Furthermore, Tables 4 and 5 depict the baseline clinical and echocardiography parameters for the high and low mobility groups, respectively. Hereby, no significant differences regarding thromboembolism risk (CHA2DS2-VASc 4.1 ± 1.6 vs. 4.1 ± 1.4, *p* = 0.99), bleeding risks (HAS-BLED 3.0 ± 1.1 vs. 2.6 ± 0.9, *p* = 0.31) and medical therapy with both NOAC (44.4 vs. 47.1%, *p* = 1.00) and OAC (66.7% vs. 76.5%, *p* = 0.71) were seen in patients with high vs. low thrombus mobility. Patients in whom thrombi resolved were more often in the group with high baseline thrombus mobility (*p* = 0.02). Furthermore, a trend to detect larger LAA thrombus (2.3 ± 1.0 mm vs. 1.7 ± 0.8 mm, *p* = 0.08) and higher peak LAA strain values (− 2.9 ± 1.4% vs. − 1.8 ± 1.5%, *p* = 0.02) in the high mobility group was observed. Of note similar LAA peak emptying velocities (25.9 ± 8.3 cm/s vs. 23.1 ± 13.8 cm/s, *p* = 0.46) and LAA volumes (6.7 ± 3.9 mL vs. 6.9 ± 3.3 mL, *p* = 0.87) were found for both groups (Table 5).

**Table 4:** Clinical baseline characteristics according to thrombus mobility.

Parameter	Total	Higher mobility	Lower mobility	*p*
*N*	35	18	17	
Age (years)	72.9 ± 14.1	71.2 ± 13.6	74.6 ± 14.7	0.49
Men (abs./%)	19 (54.3)	10 (55.6)	9 (52.9)	1.00
Thrombus resolution				0.02
Responder	18 (51.4)	13 (72.2)	5 (29.4)	
Non-responder	17 (48.6)	5 (27.8)	12 (70.5)	
eGFR (mg/dL)	50.5 ± 18.7	49.7 ± 17.4	51.4 ± 20.5	0.79
Renal insufficiency^a^ (abs./%)	7 (20.0)	4 (23.5)	3 (17.6)	1.00
Type of AF				
Paroxysmal (abs./%)	18 (51.4)	7 (38.8)	11 (64.7)	0.18
Persistent (abs./%)	14 (40.0)	9 (50.0)	5 (29.4)	0.31
Permanent (abs./%)	3 (8.6)	2 (11.1)	1 (5.9)	1.00
Diabetes (abs./%)	5 (14.3)	3 (16.7)	2 (11.8)	1.00
Hypertension (abs./%)	31 (88.6)	16 (88.9)	15 (88.2)	1.00
Coronary artery disease (abs./%)	21 (60.0)	12 (66.7)	9 (52.9)	0.50
Prior MI (abs./%)	16 (45.7)	8 (44.4)	8 (47.1)	1.00
Prior stroke (abs./%)	5 (14.3)	3 (16.7)	2 (11.8)	1.00
Prior peripheral embolism (abs./%)	1 (2.9)	0 (0.0)	1 (5.9)	0.49
Cardiomyopathy (abs./%)	11 (31.4)	7 (38.9)	4 (23.5)	0.47
Ischemic (abs./%)	9 (25.7)	6 (33.3)	3 (17.6)	0.57
Dilatative (abs./%)	2 (5.7)	1 (5.6)	1 (5.9)	1.00
CHA2DS2-VASc score	4.1 ± 1.5	4.1 ± 1.6	4.1 ± 1.4	0.99
HAS-BLED score	2.8 ± 1.0	3.0 ± 1.1	2.6 ± 0.9	0.31
Medical therapy at baseline				
NOAC (abs./%)	16 (45.7)	8 (44.4)	8 (47.1)	1.00
Any OAC (abs./%)	25 (71.4)	12 (66.7)	13 (76.5)	0.71

^a^Egfr < 30 mL/min

**Table 5:** Echocardiographic baseline characteristics according to thrombus mobility.

Parameter	Total	Higher mobility	Lower mobility	*p*
*N*	35	18	17	
Left ventricular end-diastolic volume (mL)	135.7 ± 77.2	148.6 ± 94.0	122.0 ± 53.7	0.33
Left ventricular end-systolic volume (mL)	87.8 ± 67.1	98.1 ± 81.0	76.7 ± 48.6	0.37
Left ventricular end-diastolic diameter	6.0 ± 1.3	5.99 ± 0.96	6.05 ± 1.64	0.91
Left Ventricular Ejection Fraction (%)	41.7 ± 14.4	41.5 ± 13.7	41.9 ± 15.5	0.94
Left atrial end-diastolic volume (mL)	76.3 ± 33.2	83.0 ± 32.9	69.0 ± 32.9	0.22
E-wave (cm/s)	94.5 ± 40.4	102.6 ± 38.5	83.8 ± 41.9	0.23
E’-wave (cm/s)	4.9 ± 2.5	5.4 ± 2.5	4.3 ± 2.5	0.31
*E*/*E*′	19.7 ± 8.5	16.8 ± 3.6	23.0 ± 11.2	0.11
LAA specific parameters				
LAA Volume (mL)	6.8 ± 3.6	6.7 ± 3.9	6.9 ± 3.3	0.87
LAA thrombus	1.6 ± 0.6	1.7 ± 0.6	1.5 ± 0.7	0.40
Minimum diameter (mm)	1.2 ± 0.6	1.1 ± 0.4	1.3 ± 0.8	0.36
Maximum diameter (mm)	2.0 ± 1.0	2.3 ± 1.0	1.7 ± 0.8	0.08
Time till resolution (days)	99.2 ± 71.8	103.4 ± 77.4	88.4 ± 61.3	0.70
Peak emptying velocity (cm/s)	24.5 ± 11.2	25.9 ± 8.3	23.1 ± 13.8	0.46
Maximum LAA longitudinal strain (%)	3.2 ± 2.6	3.1 ± 2.6	2.8 ± 2.6	0.85
Minimum LAA longitudinal strain (%)	− 2.3 ± 1.6	− 2.9 ± 1.4	− 1.8 ± 1.5	0.29
Mean thrombi mobility (mm)	0.25 ± 0.13	0.35 ± 0.11	0.14 ± 0.08	<0.01
Spontaneous echo contrast (abs./%)	33 (94.3)	18 (100.0)	15 (88.2)	0.22
Mitral regurgitation (abs./%)	33 (94.3)	18 (100.0)	15 (88.2)	0.21
≥ Moderate (abs./%)	21 (60.0)	11 (61.1)	10 (58.9)	1.00
Mitral stenosis (abs./%)	6 (17.1)	1 (5.6)	5 (29.4)	0.09
≥ Moderate (abs./%)	2 (5.7)	0 (0.0)	2 (11.8)	0.23
Screening time between TEE exams	51.0 ± 14.4	51.8 ± 18.6	50.1 ± 8.4	0.74

## Discussion

To the best of our knowledge, this is the first study to evaluate LAA thrombus mobility extracted from echocardiography and its role in the context of LAA thrombus resolution. The major findings of this study can be summarized as follows: (i) tracking of the LAA and LAA thrombus is feasible, and (ii) higher LAA contraction and thrombus mobility was found for patients with thrombus resolution (i.e. responder group) with statistically significant differences compared to patients with thrombus persistence (i.e. non-responder group).

In clinical routine, prediction or estimation of the risk for thromboembolism in patients with atrial fibrillation is usually based on clinical variables, such as the CHA2DS2-VASC score.^8^ In addition to these risk scores, several echocardiographic markers including LAA dimensions and peak emptying velocities,^1,15^ as well as LAA strain^11,20^ have recently been identified to be of prognostic value in the prediction of stroke risk in the context of atrial fibrillation. While these markers take into account LAA size and left atrial mechanics, little is known about the relevance of the constitution or mobility of LAA thrombi. Differently, while several researchers are currently exploring the potential of numerical simulation tools through computational fluid dynamics applied to the LA and LAA anatomy to predict the thrombosis risk,^5,6,28^ so far, no study with large populations has been performed to objectively quantify thrombus mobility and its potential clinical role.

Generally, it is intuitive to assume that higher mobility of LAA thrombi may be associated with an increased risk of embolization, as has been suggested in other contexts. For example, higher mobility of vegetations in infective endocarditis appears to be associated with an increased risk of embolization.^4^ At the same time, is also conceivable, that thrombus mobility may reflect efficacy of treatment in patients receiving oral anticoagulation for LAA thrombus. Whereas semiquantitative assessment of mobility is possible, we describe in this study a quantitative, reproduceable method of evaluating thrombus mobility. While the LAA was individually evaluated to estimate the standard strain curve, the thrombus mobility was quantified by fusing both LAA and thrombus tracking results to decompose the different motion components. The obtained results corroborated that the described tracking pipeline is feasible for the LAA and the thrombus, simply requiring the initial setting of few parameters in the tracking toolbox. When comparing both dynamic parameters, a larger separation between classes was found for the thrombi. Indeed, the obtained result was expected and can be explained by the computation strategy. While the LAA strain is computed based on the full extension of the anatomy, being therefore a global indicator of the full LAA dynamics, the thrombi mobility is localized and focused on the anomaly only. Although not explored in this study, we believe that segmental strain analysis as performed by Ref. 13, where only the segments around the thrombus are evaluated, could indeed be an interesting approach to increase the performance of the strain analysis and approximate its results to the thrombus mobility study. Nevertheless, as well reported in the literature,^2^ segmental analysis in ultrasound images is challenging and unfeasible in specific cases due to the low image quality.

As a result of our study, baseline thrombus mobility at the time point of diagnosis was significantly higher in patients in whom these thrombi eventually resolved. Additionally, we identified LAA strain and peak emptying velocities to be higher in these patients. Other factors, such as the type of anticoagulation, as well as markers of left ventricular systolic or diastolic function or the type of atrial fibrillation were not different between the groups, suggesting an independent role of LAA mechanics as well as thrombus mobility. While multivariable analysis did not reveal an independent predictive value of thrombus mobility, most likely due to limited cohort size, ROC analysis regarding thrombus resolution was highest for thrombus mobility and peak emptying velocity, further highlighting the role of these factors.

Unfortunately, the limited size of our cohort did not allow for the assessment of clinical outcome parameters such as stroke or systemic embolism. However, overall, our results suggest that LAA function loss is more severe in the non-responder group and it may work as a relevant clinical indicator to correctly stratify the patient’s risk. In this context, our findings are in line with the previous study of Karabay et al.,^12^ where a significant reduction of LAA strain was found in the presence of LAA thrombus. Of note, our mean peak LAA strain was comparable to those described in the literature, indicating the reproducibility of our findings.^11,20^

To promote the clinical use of the proposed pipeline, we have developed a software interface to simplify the interaction between the operator and the tracking method. Currently, the software is semi-automatic, requiring the manual delineation of both LAA and thrombus at one cardiac cycle through a few clicks. Our software’s workflow is comparable to commercial speckle tracking algorithms, which are routinely used in clinical practice to study LV dynamics. Moreover, although not studied in this specific article, our software is suitable for the clinical evaluation of the LAA in different echocardiographic acquisition protocols, including transthoracic (TTE) and intracardiac echocardiography (ICE). Indeed, both TTE and ICE show a similar image appearance when compared to the tested TEE (including the LAA anatomy), simply modifying the spatial anatomical representation on the image due to the variation of the probe’s position. Since our tracking method is always computed through a set of anatomical ROIs defined around the manually delineated contour, it is non-sensitive to spatial variations of the LAA site, making it therefore compatible with the abovementioned echocardiographic protocols.

Concerning the software workflow, after loading the ultrasound sequence, the proposed dynamic parameters are computed in less than 25 s (the manual segmentation took ~19.85 ± 4.56 s, while the tracking method required < 2 s), not negatively affecting the screening time and allowing online evaluation throughout the ultrasound imaging acquisition. Moreover, it does not have any relevant computational requirements. All presented results were computed with a standard laptop (Intel (R) i7 CPU at 2.8 GHz and 16 GB of RAM). Thus, the software integration in the clinical routine is feasible and envisioned in future work. Currently, we are exploring recent application programming interfaces (API) provided by US manufacturers to directly integrate our software as a third-party application inside the scanner, allowing deployment of our software into the scanner as an external plugin and direct access to the ultrasound images without any data conversion/exportation stages. Additionally, we are working on the implementation of a fully automatic software version. For that, we are adapting our previously described 3D LAA segmentation method^22^ for 2D sequences.

Although not explored in this study, the proposed tracking methodology is versatile and could be relevant in other clinical aspects. Until now, no study explored the tracking of occluded anatomies in order to estimate relevant clinical indexes to predict device-related thrombus.^25^ Here, the evaluation of the LAA dynamics after implantation and even local assessment of the left atrial body motion around the LAA could be a potential indicator of thrombus risk. Additionally, the current methodology has not any specific anatomical constraint and it could be expanded to other types of intracardiac structures (e.g. left ventricular thrombus), potentially identifying relevant mechanistic characteristics of this pathology. Its application for the identification of other potential embolic predictors (e.g. endocarditis and vegetations mobility) is also conceivable.

### Study Limitations

First, all the obtained results were obtained in a small population, requiring further study in a larger clinical database to evaluate the real performance of the described indexes. Therefore, all the described results must be analyzed as preliminary, and all the reported observations/results require further research. Moreover, concerning the computation of the thrombus mobility, full compliance of the thrombus with the LAA was assumed. That assumption may result in sub-optimal results in cases of extremely large LAA with freely floating LAA thrombi, requiring further experiments to identify potential limitations of this technique.

Second, it was a single-center study focused on a specific population. Further studies to evaluate the method’s performance in a larger multi-center database is required to validate its real performance.

Third, the study was enrolled in a selective cohort composed of patients with a high risk for thromboembolism and a moderate risk for bleeding. Here, we would like to emphasize that the selection of patients with a high risk for thromboembolism is explained by the higher likelihood to develop a thrombus. Finding evidence of thrombus in patients with low/moderate thromboembolic risk is less likely, as risk factors for thrombus development are less.

Fourth, the current study was performed in bidimensional images, which was already previously described as sub-optimal practice^23^ since it may suffer from out-plane motion artifacts. Moreover, Doppler tissue imaging, which would allow the extraction of the maximal velocity during the atrial contraction, was not routinely performed.

#### Conclusions

In summary, tracking of LAA thrombus mobility was feasible from two dimensional echocardiographic images. Higher LAA contraction and thrombus mobility were found for patients in whom thrombi resolved (responder group) with statistically significant differences against those with persistent thrombus (non-responder group). In our study, mobility of solid LAA thrombus was identified as a potential novel echocardiographic parameter to predict resolution. In any case, further confirmatory analyses are needed to establish the clinical relevance of our proposed technique.

## Supplementary Information

Below is the link to the electronic supplementary material.

Supplementary file1 (MP4 1349 kb)

Supplementary file2 (MP4 1157 kb)

Supplementary file3 (MP4 1427 kb)

Supplementary file4 (MP4 749 kb)

Supplementary file5 (MP4 1592 kb)

Supplementary file6 (PDF 211 kb)

## Data Availability

The data underlying this article will be shared on reasonable request to the corresponding author.
