# Supplementary material for: Assessment of LAA Strain and Thrombus Mobility and Its Impact on Thrombus Resolution—Added-Value of a Novel Echocardiographic Thrombus Tracking Method
Source: Cardiovasc Eng Technol. 2022 May 13;13(6):950–60. doi: 10.1007/s13239-022-00629-z (PMC9750899; doi:10.1007/s13239-022-00629-z)
Supplement: Supplementary file 6 — Supplementary file6 (PDF 211 kb) [file 13239_2022_629_MOESM6_ESM.pdf]

Logistic Regression

Case Processing Summary

Unweighted Cases[a]		N	Percent
Selected Cases	Included in Analysis	29	80,6
	Missing Cases	7	19,4
	Total	36	100,0
Unselected Cases		0	,0
Total		36	100,0

a. If weight is in effect, see classification table for the total number of cases.

Dependent Variable Encoding

Original Value	Internal Value
non-resp	0
resp	1

Block 0: Beginning Block

Iteration History[a,b,c]

Iteration		-2 Log likelihood	Coefficients
			Constant
Step 0	1	40,168	,069
	2	40,168	,069

a. Constant is included in the model.

b. Initial -2 Log Likelihood: 40,168

c. Estimation terminated at iteration number 2 because parameter estimates changed by less than ,001.

Classification Table[a,b]

Observed			Predicted		
			Group		Percentage Correct
			non-resp	resp	
Step 0	Group	non-resp	0	14	,0
		resp	0	15	100,0
	Overall Percentage				51,7

a. Constant is included in the model.

b. The cut value is ,500

Variables in the Equation

		B	S.E.	Wald	df	Sig.	Exp(B)
Step 0	Constant	,069	,372	,034	1	,853	1,071

Variables not in the Equation

			Score	df	Sig.
Step 0	Variables	Thrombi_Mobility	12,531	1	,000
		E_E	1,102	1	,294
		LAA_Volume	8,274	1	,004
		Peak_empty_velocity	11,898	1	,001
		Time_Between_TEE_exams	,434	1	,510
	Overall Statistics		21,096	5	,001

Block 1: Method = Enter

Iteration History[a,b,c,d]

Iteration		-2 Log likelihood	Coefficients			
			Constant	Thrombi_Mobility	E_E	LAA_Volume
Step 1	1	15,915	-2,452	8,001	-,004	-,123
	2	10,953	-4,020	14,207	,001	-,214
	3	8,377	-6,515	22,840	,018	-,305
	4	6,264	-12,145	38,399	,033	-,401
	5	4,461	-21,423	65,762	,058	-,649
	6	2,192	-39,459	129,177	,101	-1,622
	7	,775	-65,191	220,313	,142	-2,952
	8	,275	-90,425	308,797	,183	-4,202
	9	,100	-114,917	394,461	,223	-5,404
	10	,036	-139,098	478,967	,263	-6,588
	11	,013	-163,160	563,027	,303	-7,766
	12	,005	-187,176	646,919	,343	-8,940
	13	,002	-211,176	730,748	,383	-10,114
	14	,001	-235,169	814,554	,423	-11,288
	15	,000	-259,160	898,350	,463	-12,461
	16	,000	-283,150	982,144	,503	-13,634
	17	,000	-307,139	1065,936	,543	-14,807
	18	,000	-331,129	1149,728	,583	-15,980
	19	,000	-355,118	1233,519	,623	-17,153
	20	,000	-379,108	1317,311	,663	-18,327

Iteration History[a,b,c,d]

Iteration		Coefficients	
		Peak_empty_velocity	Time_Between_TEE_exams
Step 1	1	,067	-,005
	2	,106	-,011
	3	,146	-,014
	4	,205	,009
	5	,294	,050
	6	,384	,171
	7	,556	,325
	8	,742	,468
	9	,925	,604
	10	1,107	,738
	11	1,288	,872
	12	1,469	1,005
	13	1,650	1,138
	14	1,831	1,271
	15	2,012	1,404
	16	2,193	1,537
	17	2,374	1,670
	18	2,555	1,803
	19	2,736	1,936
	20	2,917	2,069

a. Method: Enter

b. Constant is included in the model.

c. Initial -2 Log Likelihood: 40,168

d. Estimation terminated at iteration number 20 because maximum iterations has been reached. Final solution cannot be found.

Omnibus Tests of Model Coefficients

		Chi-square	df	Sig.
Step 1	Step	40,168	5	,000
	Block	40,168	5	,000
	Model	40,168	5	,000

Model Summary

Step	-2 Log likelihood	Cox & Snell R Square	Nagelkerke R Square
1	,000[a]	,750	1,000

a. Estimation terminated at iteration number 20 because maximum iterations has been reached. Final solution cannot be found.

Hosmer and Lemeshow Test

Step	Chi-square	df	Sig.
1	,000	5	1,000

Contingency Table for Hosmer and Lemeshow Test

		Group = non-resp		Group = resp		Total
		Observed	Expected	Observed	Expected	
Step 1	1	3	3,000	0	,000	3
	2	3	3,000	0	,000	3
	3	3	3,000	0	,000	3
	4	3	3,000	0	,000	3
	5	2	2,000	1	1,000	3
	6	0	,000	3	3,000	3
	7	0	,000	11	11,000	11

Classification Table[a]

Observed			Predicted		
			Group		Percentage Correct
			non-resp	resp	
Step 1	Group	non-resp	14	0	100,0
		resp	0	15	100,0
	Overall Percentage				100,0

a. The cut value is ,500

Variables in the Equation

		B	S.E.	Wald	df	Sig.
Step 1[a]	Thrombi_Mobility	1317,311	93587,874	,000	1	,989
	E_E	,663	170,146	,000	1	,997
	LAA_Volume	-18,327	1460,795	,000	1	,990
	Peak_empty_velocity	2,917	324,769	,000	1	,993
	Time_Between_TEE_exams	2,069	191,787	,000	1	,991
	Constant	-379,108	28865,727	,000	1	,990

Variables in the Equation

		Exp(B)	95% C.I.for EXP(B)	
			Lower	Upper
Step 1[a]	Thrombi_Mobility	.	,000	.
	E_E	1,941	,000	1,308E+145
	LAA_Volume	,000	,000	.
	Peak_empty_velocity	18,478	,000	5,136E+277
	Time_Between_TEE_exams	7,915	,000	1,407E+164
	Constant	,000		

a. Variable(s) entered on step 1: Thrombi_Mobility, E_E, LAA_Volume, Peak_empty_velocity, Time_Between_TEE_exams.

Correlation Matrix

		Constant	Thrombi_Mobility	E_E	LAA_Volume
Step 1	Constant	1,000	-,931	-,493	,710
	Thrombi_Mobility	-,931	1,000	,305	-,867
	E_E	-,493	,305	1,000	-,197
	LAA_Volume	,710	-,867	-,197	1,000
	Peak_empty_velocity	-,760	,569	,506	-,252
	Time_Between_TEE_exams	-,774	,779	,184	-,811

Correlation Matrix

		Peak_empty_velocity	Time_Between_TEE_exams
Step 1	Constant	-,760	-,774
	Thrombi_Mobility	,569	,779
	E_E	,506	,184
	LAA_Volume	-,252	-,811
	Peak_empty_velocity	1,000	,283
	Time_Between_TEE_exams	,283	1,000

Nominal Regression

Case Processing Summary

		N	Marginal Percentage
Group	non-resp	14	48,3%
	resp	15	51,7%
Valid		29	100,0%
Missing		7	
Total		36	
Subpopulation		29[a]	

a. The dependent variable has only one value observed in 29 (100,0%) subpopulations.

Model Fitting Information

Model	Model Fitting Criteria			Likelihood Ratio Tests		
	AIC	BIC	-2 Log Likelihood	Chi-Square	df	Sig.
Intercept Only	42,168	43,535	40,168			
Final	12,000	20,204	,000	40,168	5	,000

Goodness-of-Fit

	Chi-Square	df	Sig.
Pearson	,000	23	1,000
Deviance	,000	23	1,000

Pseudo R-Square

Cox and Snell	,750
Nagelkerke	1,000
McFadden	1,000

Likelihood Ratio Tests

Effect	Model Fitting Criteria			Likelihood Ratio Tests	
	AIC of Reduced Model	BIC of Reduced Model	-2 Log Likelihood of Reduced Model	Chi-Square	df
Intercept	20,513	27,350	10,513	10,513	1
Thrombi_Mobility	28,712	35,549	18,712	18,712	1
E_E	10,000	16,836	,000[a]	,000	1
LAA_Volume	10,000	16,837	,000[a]	,000	1
Peak_empty_velocity	19,430	26,267	9,430	9,430	1
Time_Between_TEE_exams	10,000	16,836	,000[a]	,000	1

Likelihood Ratio Tests

Effect	Likelihood Ratio Tests
	Sig.
Intercept	,001
Thrombi_Mobility	,000
E_E	,999
LAA_Volume	,989
Peak_empty_velocity	,002
Time_Between_TEE_exams	,999

The chi-square statistic is the difference in -2 log-likelihoods between the final model and a reduced model. The reduced model is formed by omitting an effect from the final model. The null hypothesis is that all parameters of that effect are 0.

a. The log-likelihood values are approaching zero. There may be a complete separation in the data. The maximum likelihood estimates do not exist.

Parameter Estimates

Group[a]		B	Std. Error	Wald	df	Sig.
non-resp	Intercept	379,150	28891,567	,000	1	,990
	Thrombi_Mobility	-1317,461	93672,342	,000	1	,989
	E_E	-,663	170,303	,000	1	,997
	LAA_Volume	18,329	1462,153	,000	1	,990
	Peak_empty_velocity	-2,917	325,014	,000	1	,993
	Time_Between_TEE_exams	-2,069	191,956	,000	1	,991

Parameter Estimates

Group[a]		Exp(B)	95% Confidence Interval for Exp(B)	
			Lower Bound	Upper Bound
non-resp	Intercept			
	Thrombi_Mobility	,000	,000	.
	E_E	,515	5,619E-146	4,724E+144
	LAA_Volume	91223925,187	,000	.
	Peak_empty_velocity	,054	1,206E-278	2,428E+275
	Time_Between_TEE_exams	,126	5,112E-165	3,121E+162

a. The reference category is: resp.

b. Floating point overflow occurred while computing this statistic. Its value is therefore set to system missing.

Classification

Observed	Predicted		
	non-resp	resp	Percent Correct
non-resp	14	0	100,0%
resp	0	15	100,0%
Overall Percentage	48,3%	51,7%	100,0%
